# Pulmonary Rehabilitation for Exercise Tolerance and Quality of Life in IPF Patients: A Systematic Review and Meta-Analysis

**DOI:** 10.1155/2019/8498603

**Published:** 2019-03-21

**Authors:** Xueqing Yu, Xuanlin Li, Liaoyao Wang, Ran Liu, Yang Xie, Suyun Li, Jiansheng Li

**Affiliations:** ^1^Collaborative Innovation Center for Respiratory Disease Diagnosis and Treatment & Chinese Medicine Development of Henan Province, Henan University of Chinese Medicine, Zhengzhou, Henan 450046, China; ^2^Henan Key Laboratory of Chinese Medicine for Respiratory Disease, Henan University of Chinese Medicine, Zhengzhou, Henan 450046, China; ^3^Department of Respiratory Diseases, The First Affiliated Hospital of Henan University of Chinese Medicine, Zhengzhou, Henan 450000, China

## Abstract

**Objective:**

The aim of this study is to evaluate the efficacy and safety of pulmonary rehabilitation (PR) in patients with idiopathic pulmonary fibrosis (IPF).* Method*s. Embase, PubMed, Cochrane Library, China National Knowledge Infrastructure (CNKI), Chongqing VIP (CQVIP), Wanfang Data, and Chinese Biomedical Literature Database (SinoMed) were comprehensively searched. Randomized controlled trials (RCTs) that investigated the effects of PR for IPF patients were included. Literature selection and data extraction were conducted by two review authors independently. The Cochrane Collaboration's Risk of Bias tool and RevMan software (version 5.3) were used to evaluate the quality of studies and conduct statistical analysis, respectively.* Resul*ts. Seven studies (190 participants) were included. PR had a significant effect on six-minute walk distance (6MWD) (MD:48.60; 95%CI: 29.03 to 68.18;* Z*=4.87,* P*<0.00001), and 6MWD was improved more in subgroup analysis including studies conducted in Asia (MD: 53.62; 95%CI: 30.48 to 76.66;* Z*=4.54,* P*<0.00001) and Europe (MD:54.10; 95% CI: 26.65 to 101.56;* Z*=2.23,* P*=0.03). Forced vital capacity (FVC%) was higher (MD: 3.69; 95%CI: 0.16 to 7.23;* Z*=2.05,* P*=0.04). St. George's Respiratory Questionnaire (SGRQ)/IPF-specific SGRQ (SGRQ-I) total score was lower (MD: -7.87; 95% CI: -11.44 to -4.30;* Z*=4.32,* P*<0.0001). No significant effects were found for lung diffusing capacity determined by the single-breath technique (DLCO%) (MD: 3.02; 95%CI: -0.38 to 6.42;* Z*=1.74,* P*=0.08).

**Conclusions:**

This study suggests that PR may enhance exercise capacity and improve quality of life in IPF patients. Besides, PR may also delay the decline of lung function of patients with IPF. However, further research should more fully assess the efficacy and safety of PR for IPF.

## 1. Introduction

Idiopathic pulmonary fibrosis (IPF) is a chronic, progressive, and the most common subtype of interstitial lung disease (ILD) characterized by dyspnea and progressive deterioration of lung function [1, 2]. In recent years, the incidence of IPF has been increasing worldwide, especially in Europe and North America and other developed countries. In these ones, the incidence was conservatively estimated to be 3 to 9 cases per 100,000 residents per year [3, 4]. The median survival of IPF patients is only 2-3 years [5, 6], and acute exacerbations and complications may lead to hospitalization and death, resulting in a significant economic and health care burden [7–9]. Although pharmacological therapy can relieve the symptoms and delay the decline of lung function of patients with IPF, the overall efficacy is still not satisfactory, and its cost and adverse effects cannot be ignored [10–12].

Pulmonary rehabilitation (PR) is one of the most popular treatments for nonpharmacological therapy, which has been recommended by guidelines for the treatment of patients with IPF; however, the evidence is still inconclusive [1, 13]. Recently, two representative meta-analyses have introduced the application of PR in patients with IPF [14, 15]. However, the latest meta-analysis included other types of ILD patients, which affected the evaluation of PR efficacy to some extent [14]. In addition, the most recent study included in another meta-analysis was published in 2015, since then a number of randomized controlled trials (RCTs) have been published [15].

Thus, it is necessary to recollect and analyze the evidence to evaluate the efficacy and safety of PR in IPF treatment.

## 2. Methods and Analysis

### 2.1. Literature Search

In this study, the following databases: Embase, PubMed, Cochrane Library, China National Knowledge Infrastructure (CNKI), Chongqing VIP (CQVIP), Wanfang Data, and Chinese Biomedical Literature Database (SinoMed) were comprehensively searched as of July 2, 2018. We developed systematically search strategies for each electronic database without language limitations to attempt to identify all eligible studies. Medical subject heading (MESH) terms and key words regarding the participant and intervention included (“Pulmonary Fibroses, Idiopathic” OR “Idiopathic Pulmonary Fibrosis” OR “IPF”) AND (“pulmonary rehabilitation” OR “rehabilitation therapy” OR “exercise therapy” OR “rehabilitation training” OR “rehabilitation program” OR “exercise training” OR “exercise program” OR “resistance training” OR “physical training”) AND (“randomized controlled trials” OR “RCTs” OR “controlled clinical trial” OR “randomization”). Reference lists of studies and relevant systematic reviews were manually screened to identify further eligible researches. Two reviewers (Xueqing Yu and Xuanlin Li) performed the literature search independently. Any discrepancy was resolved by arbitration or discussion with a third reviewer (Jiansheng Li).

### 2.2. Inclusion and Exclusion Criteria

The following inclusion criteria were applied: (I) IPF participants should be diagnosed according to previous or current guidelines for the American Thoracic Society (ATS)/European Respiratory Society (ERS). (II) The experimental and control groups should have same interventions, with the exception of PR programs in the experimental group. PR was defined as a comprehensive program composed at least one of exercise training, educational lectures, or self-administered. (III) The outcomes of interest were exercise capacity (measured by 6MWT/6MWD), lung function (measured by FVC% and DLCO% predicted), and quality of life (measured by SGRQ/IPF-specific SGRQ) as well as adverse events. (IV) RCTs evaluate the effects of PR on IPF patients. Exclusion criteria are as follows: (I) Not RCTs (retrospective, observational, cohort as well as case control studies). (II) Conference abstracts with no corresponding full article published in journal. (III) Duplicate publications. Data were only extracted from the study with the most up-to-date information. (IV) No outcomes of interest. (V) Study protocol.

### 2.3. Study Selection

The study selection was performed by two coauthors (Liaoyao Wang and Ran Liu) in two phases to determine which articles are suitable. At first, duplicated and nonrelevant studies were discarded by examining titles and abstracts. Secondly, in accordance with the study inclusion criteria and exclusion criteria, eligible studies were extracted by reviewing full-text articles. A third coauthor (Yang Xie) acted as an arbiter who resolved any arguments that occurred.

### 2.4. Data Extraction

Two coauthors (Xuanlin Li and Yang Xie) independently extracted data from the eligible trials using standardized data extraction forms, including information of authors, year of publication, experiment design, characteristics of participants, course of treatment, interventions, comparators, and outcomes, with a third review author (Suyun Li) acting as an arbiter when disagreements occurred.

### 2.5. Assessment of Risk of Bias

Quality assessments were performed with the Cochrane risk of bias tool [23], which contains 7 items. Two coauthors (Xuanlin Li and Liaoyao Wang) independently assessed and scored each research. In the event of disagreement, they reviewed the original article and discussed with another author (Suyun Li) to reach a consensus.

### 2.6. Statistical Analysis

All analyses were accomplished by Review Manager 5.3 software (RevMan; The Nordic Cochrane Centre, The Cochrane Collaboration, Copenhagen, 2014) [24]. The summary effect size was estimated by using odds ratio (OR) with 95% confidence intervals (CI) for dichotomous outcomes and mean difference (MD) with 95% CI for continuous outcomes. If the same outcome was measured with different methods or scales, we calculated standardized mean differences (SMD) instead of MD. A* χ*^2^ test was used to estimating heterogeneity of both the OR and MD. Further analysis was performed using the I^2^ test. The* χ*^2^ test with P<0.1 and I^2^>50% indicated significant heterogeneity, and a random-effect model was used to combine the results, whereas a fixed-effect model was chosen. Sensitivity analysis involves deleting each study separately to assess the quality and consistency of the results. If the meta-analysis included more than 10 studies, we would investigate publication bias by funnel plots. If the data permitted, we conducted subgroup analysis to assess whether the treatment effects were different in different situation.

## 3. Results

### 3.1. Literature Search and Study Selection

**Figure 1:**
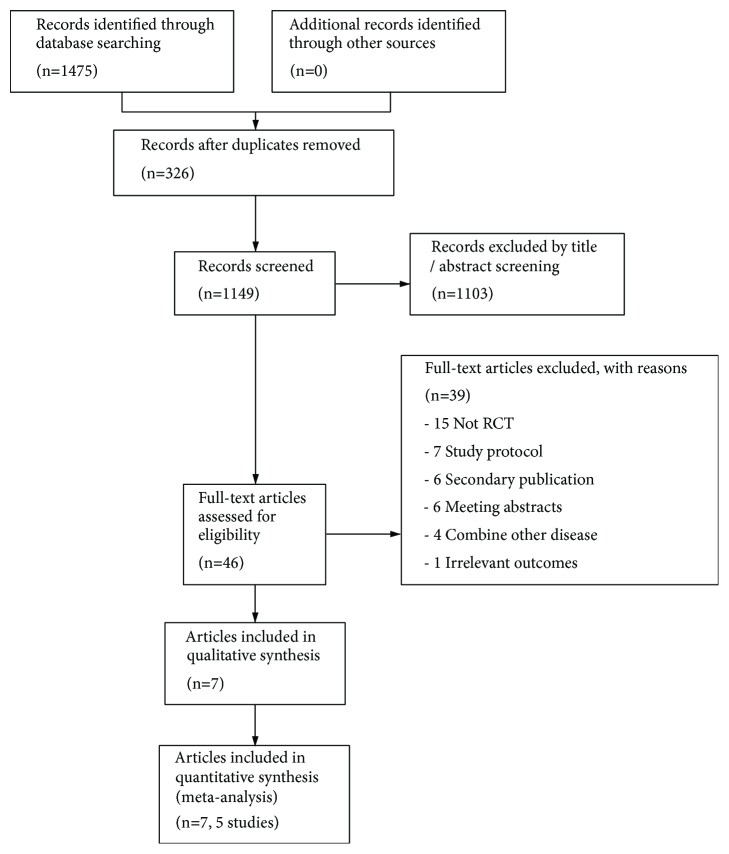
Study flow diagram.

A total of 1475 articles were identified through initial literature searches, of which 326 were duplicates. After reviewing the titles and/or abstracts, 46 potentially relevant full studies remained for further assessment. After reviewing the full papers to obtain additional details, 39 selected articles were excluded for following reasons: (1) not RCTs (n=15), no interest outcomes reported (n=1), meeting abstracts (n=6), and secondary reports (n=6) as well as study protocol (n=7). Seven articles and 5 studies were included in the final analysis. The study selection process is shown in Figure 1.

### 3.2. Characteristics of the Included Studies

All included studies were RCTs, published from 2008 to 2016. The five trials were conducted in five different countries in three continents. The number of participants is 190: 95 patients were assigned to PR group, while the others were administered to control group. The number of participants in the included studies ranged from 21 to 32 and 10 to 12 weeks of PR programs were performed. The PR program at least included one of exercise training, educational lectures, or self-administered.  Table 1 summarizes a detailed description of the characteristics of included studies.

### 3.3. Assessment of Risk of Bias

**Figure 2:**
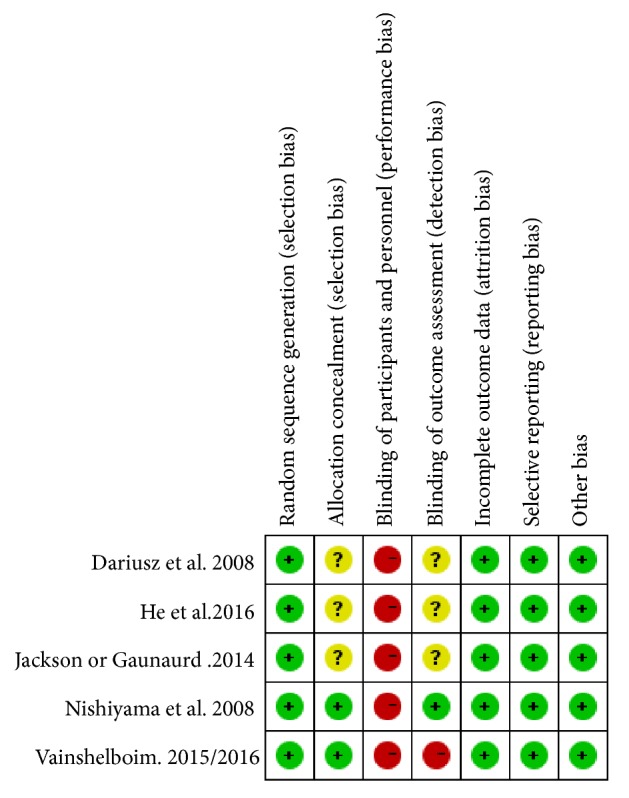
Risk of bias summary: review authors' judgements about each risk of bias item for each included study.

**Figure 3:**
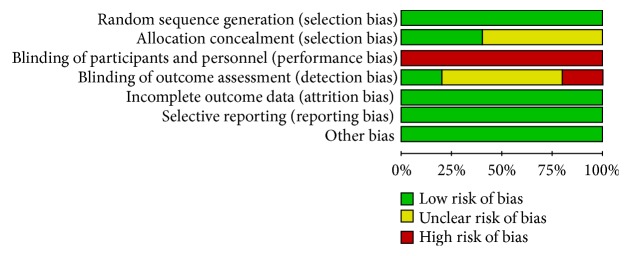
Risk of bias graph: review authors' judgements about each risk of bias item presented as percentages across all included studies.

We determined the Cochrane “risk of bias” score for each study. All of the included studies had similar group characteristics at baseline and randomized, but all included studies did not blind their patients because of the nature of the intervention. Two studies reported on their methods of allocation concealment, and one study reported on their methods of blinding of outcome assessment, while a study was unblinded in outcome assessment. All studies described the reasons patients withdrew from the study or fall off, but there is no intention to analysis. A detailed evaluation was provided in Figures 2 and 3.

### 3.4. Effects of Interventions

#### 3.4.1. 6MWD

**Figure 4:**
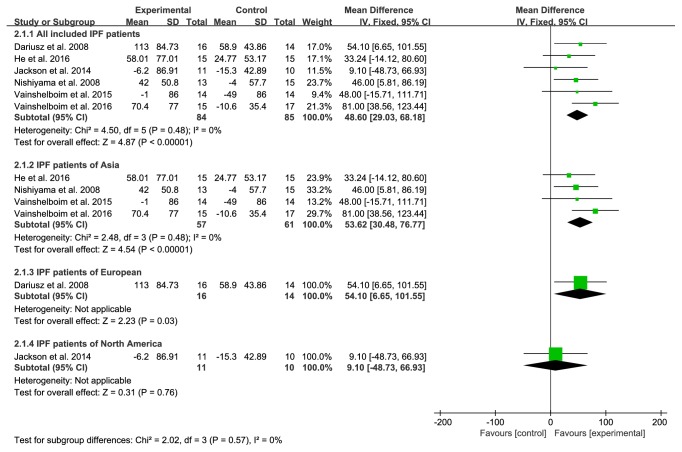
PR group versus control group, 6MWD.

Six studies provided numerical data for 6MWD and were included in the meta-analysis. There was no significant heterogeneity (*χ*^2^=2.02, P=0.57, I^2^=0%); therefore, a fixed-effects model was chosen. The analysis showed a significant treatment effect on 6MWD (MD 48.60, 95% CI: 29.03 to 68.18, P<0.00001), and 6MWD improved more in subgroup analysis including studies conducted in Asia (MD:53.62; 95% CI:30.48 to 76.66;* Z*=4.54,* P*<0.00001) and Europe (MD:54.10; 95% CI:26.65 to 101.56;* Z*=2.23,* P*=0.03) (Figure 4).

#### 3.4.2. SGRQ/SGRQ-I Total Score

**Figure 5:**
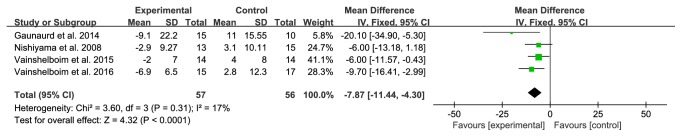
PR group versus control group, SGRQ/SGRQ-I.

Among the included studies, four studies provided complete data for SGRQ/SGRQ-I total score. No significant heterogeneity was observed (*χ*^2^=4.32, P<0.0001, I^2^=17%); therefore, a fixed-effects model was used to combine the results. A positive influence on improving quality of life was noted after the treatment (MD -7.87, 95% CI: -11.44 to -4.30, P=0.031; Figure 5).

#### 3.4.3. FVC%

**Figure 6:**
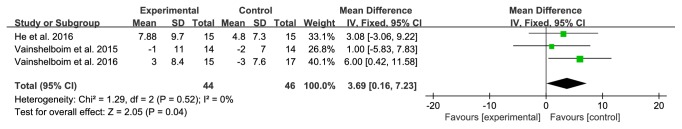
PR group versus control group, FVC%.

Among the included studies, 3 studies provided numerical data for FVC% and were included in the meta-analysis. There was no significant heterogeneity (*χ*^2^ =2.05, P=0.04, I^2^=0%); therefore, a fixed-effects model was used. The analysis showed a significant treatment effect on FVC% (MD 3.69, 95% CI: 0.16 to 7.23, P=0.52; Figure 6).

#### 3.4.4. DLCO%

**Figure 7:**
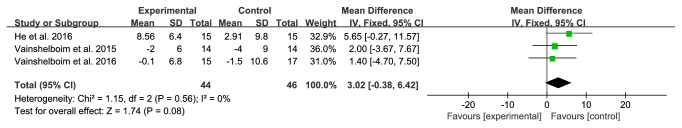
PR group versus control group, DLCO%.

Among the included studies, 3 studies provided numerical data for DLCO% and were included in the meta-analysis. The heterogeneity was not statistically significant (*χ*^2^=1.15, P=0.56, I^2^=0%); hence, a fixed-effects model was used. The analysis showed no significant improvement on DLCO% (MD 3.02, 95% CI: -0.38 to 6.42, P=0.0.56; Figure 7).

### 3.5. Adverse Effects

No adverse events were reported in all included studies [16–22].

### 3.6. Sensitivity Analysis

The data were reanalyzed by deleting each study individually. The most of outcomes yielded consistent results. However, after excluding the study conducted by He et al. 2016 on FVC%, the direction of the outcome reversed (Table 2).

## 4. Discussion

PR is a multidisciplinary and comprehensive intervention, including not only endurance training, aerobic exercise, and respiratory muscle training, but also oxygen therapy, nutritional intervention, education, self-management, etc. [25]. In recent years, PR has been widely used in the intervention and treatment of chronic respiratory diseases, playing an important role in improving the exercise capacity, health-related quality of life and breathlessness [26, 27]. IPF is a chronic respiratory disease with chronic, continuous progress and poor prognosis. This systematic review summarizes the current evidence on the efficacy and safety of PR in functional effects and quality of life in IPF patients.

According to our knowledge, there are three advantages of this systematic review. Firstly, the comprehensive literature retrieval system, the number of the research, and the number of in-taking subjects are the most of the meta-analysis of the IPF in the treatment of PR, and all the study comes from 5 countries, 3 continents, in three languages, further reducing the regional bias and language bias. Secondly, the subjects included in the study were all patients with IPF which did not include other types of ILD, and the diagnostic criteria were all in line with the relevant standards formulated by the ATS/ERS, which was conducive to the evaluation of the efficacy of PR in IPF. Thirdly, it is more reasonable and standard to include data extraction of evaluation outcome indicators.

6MWD is a reliable, effective, and reactive measure of disease status and an effective endpoint for IPF clinical trials. It has been found to help assess disease stage severity, provide information on therapeutic outcomes, and predict morbidity and mortality [28, 29]. In our review, the 6MWD of the PR group was more compared to the control group, and the MD of all IPF patients included was 48.60 meters. In the subgroup analysis, the 6MWD of IPF patients in Asia and Europe was more obvious than that of the control group, exceeding the minimal clinically important difference (MCID) of 6MWD for IPF patients about 31-46 meters [30]. This result may indicate that PR has a great potential to increase that exercise capacity of IPF patients. However, the 6MWD of IPF patients in North America showed no significant difference in efficacy between the experimental group and the control group, which may be related to the small number of included studies and samples.

The SGRQ/SGRQ-I is a useful measure of health-related quality of life in patients with IPF [31]. The SGRQ-I, revised from SGRQ to 34 items, contains the same domain and total score range. The total score of these two questionnaires is between 0 and 100, and the higher the score, the worse the quality of life related to health. In this review, the SGRQ/SGRQ-I in the PR group was lower compared to the control group, and the MD was -7.87 with a 95% CI of -11.44 to -4.30, exceeding the starting point about 4-5 points for IPF trials [32], and this might indicate the effect of PR on different aspects of health status in IPF patients.

FVC% and DLCO% are widely used in the diagnosis, classification, treatment, monitoring, and prognosis of IPF patients. In this review, FVC% and DLCO% were only available in 3 articles and showed a significant treatment effect on FVC%, but it was limited to support the effect of PR in improving lung functions in IPF patients. However, there was no statistically significant improvement in DLCO% between two groups.

## 5. Conclusions

PR can effectively improve the movement capacity and quality of life of IPF patients. In addition, PR also can improve the FVC% of IPF patients. The evidence is insufficient to support the potential to improve DLCO% in IPF patients. No serious adverse events were reported. The evidence has certain references to clinicians, patients, and health policy makers in the application of IPF. However, further high quality RCTs are required to confirm the effectiveness and safety of PR to patients with IPF.

## Figures and Tables

**Table 1:** Characteristics of the studies included in the review.

Author year	Country	Study design	No. of patients (n)	Gender (M/F)	Age	Duration	Program of PR	CG group	Outcomes
Vainshelboim et al. 2016[16]	Israel	RCTs, 2arms	32 PR 15 CG 17	PR 10/5 CG 11/6	PR 68.8±6 CG 66±9	12-week	Aerobic, Resistance, Flexibility exercise, Modes and deep breathing exercises	Regular care	FVC%%, DLCO%%, 6MWD, IPAQ SGRQ scores

Vainshelboim et al. 2015[17]	Israel	RCTs, 2arms	28 PR 14 CG 14	/	PR 68.8±6 CG 66±9	11-month follow-up	Aerobic, Resistance, Flexibility exercise, Modes and deep breathing exercises	Regular care	FVC%%, DLCO%%, 6MWD, SGRQ scores, IPAQ
He et al. 2016[18]	China	RCTs, 2arms	30 PR 15 CG 15	PR 9/6 CG 8/7	PR 64.87±7.36 CG 65.33±7.74	12-week	Cycle ergometer	Regular care	FVC%%, DLCO%%, 6MWD, TLC%, ATAQ-IPF scores
Gaunaurd et al.2014[19]	America	RCTs, 2arms	21 PR 11 CG 10	/	PR 71±6 CG 66±7	12 weeks 3-month follow-up	Educational lectures, Supervised aerobic, Strengthening exercises.	Regular care	IPF-specific SGRQ scores IPAQ, FVC%, DLCO%%
Robert et al. 2014[20]	America	RCTs, 2arms	21 PR 11 CG 10	/	PR 71±6 CG 66±7	12 weeks	Educational lectures, Treadmill walking, Semi recumbent cycling, Self-administered, Flexibility exercises, Strength training	Regular care	6MWD, TLC%
Nishiyama et al. 2008[21]	Japan	RCTs, 2arms	28, PR 13 CG 15	PR 12/1 CG 9/6	PR 68.1±8.9 CG 64.5±9.1	10 weeks	Treadmill, Cycle ergometer test, Supplemental oxygen, Elastic bands, Arm raising, Knee extensions, Educational lectures	Regular care	6MWD, SGRQ scores FVC%, FEV1, TLC, BDI
Dariusz et al. 2008[22]	Poland	RCTs, 2arms	30 PR 16 CG 14	PR 10/6 CG 9/5	PR 56.5 ± 6.5 CG 56.2 ± 7.2	12 weeks	Inspiratory muscle training	Regular care	6MWD, BDI, SF-36

Abbreviations: RCTs: randomized controlled trials; PR: pulmonary rehabilitation; CG: control group; FVC%: forced vital capacity; DLCO%: diffusion capacity for carbon monoxide; 6MWD: six-minute walk; SGRQ: St. George's Respiratory Questionnaire; IPAQ: International Physical Activity Questionnaire; IPF-specific SGRQ: St. George's Respiratory Questionnaire specific for IPF; FEV1: forced expiratory volume in 1 second, test/distance, BDI, baseline dyspnea index; SF-36: social functioning-36.

**Table 2:** Results of sensitivity analysis.

Outcomes	Deletion	Result	
6MWD	Dariusz et al. 2008	*χ* ^2^=4.44, P<0.0001, I^2^=10%	MD47.48, 95% CI: 25.99 to 68.97
	He et al. 2016	*χ* ^2^=4.01, P<0.00001, I^2^=0%	MD51.77, 95% CI: 30.27 to 73.27
	Jackson et al. 2014	*χ* ^2^=2.48, P<0.00001, I^2^=0%	MD53.72, 95% CI: 32.91 to 74.52
	Nishiyama et al. 2008	*χ* ^2^=4.48, P <0.0001, I^2^=11%	MD49.41, 95% CI: 27.00 to 71.83
	Vainshelboim et al. 2015	*χ* ^2^=4.50, P <0.00001, I^2^=11%	MD48.67, 95% CI: 28.10 to 69.24
	Vainshelboim et al. 2016	*χ* ^2^=1.66, P =0.0004, I^2^=0%	MD39.85, 95% CI: 17.79 to 61.91
SGRQ scores	Gaunaurdet al.2014	*χ* ^2^=0.82, P =0.0002, I^2^=0%	MD-7.11, 95% CI: -10.79 to -3.43
	Nishiyama et al. 2008	*χ* ^2^=3.26, P<0.0001, I^2^=39%	MD-8.48, 95% CI: -12.60 to -4.37
	Vainshelboim et al. 2015	*χ* ^2^=2.87, P=0.0001, I^2^=30%	MD-9.17, 95% CI: -13.83 to -4.52
	Vainshelboim et al. 2016	*χ* ^2^=3.20, P=0.0009, I^2^=38%	MD-7.14, 95% CI: -11.36 to -2.93
FVC%	He et al. 2016	*χ* ^2^=1.23, P=0.007, I^2^=19%	MD4.00, 95 %CI: -0.32 to8.32
	Vainshelboim et al. 2015	*χ* ^2^=0.48, P=0.003, I^2^=0%	MD4.68, 95% CI: 0.55 to 8.81
	Vainshelboim et al. 2016	*χ* ^2^=0.20, P=0.36, I^2^=0%	MD2.15, 95% CI: -2.42 to 6.72
DLCO%	He et al. 2016	*χ* ^2^=0.02, P=0.42, I^2^=0%	MD1.72, 95% CI: -2.43 to 5.87
	Vainshelboim et al. 2015	*χ* ^2^=0.98, P=0.10, I^2^=0%	MD3.59, 95% CI: - 0.66 to7.84
	Vainshelboim et al. 2016	*χ* ^2^=0.76, P=0.07, I^2^=0%	MD3.74, 95% CI: -0.35 to7.84

## Additional Points

*Registration Number*. This article is registered with PROSPERO 2018 CRD42018100911.

## Supplementary Materials

Supplementary Materials This study was conducted in accordance with the Preferred Reporting Items for Systematic Reviews and Meta-Analysis (PRISMA) criteria [33].
